# Periprosthetic supracondylar femoral fractures following total knee arthroplasty: clinical comparison and related complications of the femur plate system and retrograde-inserted supracondylar nail

**DOI:** 10.1007/s10195-014-0287-x

**Published:** 2014-04-01

**Authors:** Viral Gondalia, Duck Hyun Choi, Su Chan Lee, Chang Hyun Nam, Bo Hyun Hwang, Hye Sun Ahn, Alvin C. Ong, Ha Young Park, Kwang Am Jung

**Affiliations:** 1 Joint and Arthritis Research, Department of Orthopaedic Surgery, Himchan Hospital, 20-8, Songpa-dong, Songpa-gu, Seoul, 138-170 Korea; 2 Rothman Institute, 2500 English Creek Road, Bldg. 1300, Egg Harbor Township, NJ 08234 USA

**Keywords:** Periprosthetic fracture, Total knee arthroplasty, Femur plate system, Retrograde-inserted supracondylar nail

## Abstract

**Background:**

The purpose of this study is to analyze the clinical results and related complications of the femur plate system (FP) and the retrograde-inserted supracondylar nail (RISN).

**Materials and methods:**

The study included 42 cases of periprosthetic supracondylar femoral fractures (PSF) proximal to posterior stabilized total knee arthroplasty between 2005 and 2009. Twenty-four cases of PSF were treated with the FP, and the other 18 cases were treated with the RISN. This study cohort was divided into subgroups according to the AO classification. We retrospectively compared the clinical results between the FP and RISN group.

**Results:**

There were no significant differences between the two groups in terms of time of clinical union (*p* = 0.649). In the subgroup analysis, the mean operation time was significantly different only in subgroup A1 (*p* = 0.03). Complications were seen in 29.2 % (7/24) of patients in the FP group and 27.8 % (5/18) in the RISN group. The age during the index TKA and fracture fixation was a significant risk (*p* = 0.008) factor for complications between the two groups. No significant differences were found in the other factors between the two groups. The *p* value for operative time (*p* = 0.223), immobilization period (*p* = 0.129), ROM (*p* = 0.573), KSS (*p* = 0.379), KSS functional scores (*p* = 0.310) and time to union (*p* = 0.649).

**Conclusion:**

Clinical results did not differ according to the treatment methods used. Fixation method and fracture type did not cause an increase in the complication rate, but there was a trend toward higher non-union rates with the FP method and higher re-fracture rate with the RISN method. Noting the fact that only increasing age correlated with an increased complication rate, more careful attention should be paid to elderly patients in terms of both prevention and surgical care.

**Level of evidence:**

Level III, therapeutic study.

## Introduction

With an aging population, the numbers of total knee arthroplasties performed in patients of advanced age are on the rise. Consequently, periprosthetic supracondylar femoral fractures (PSF) are becoming more common. This is due to several factors including, but not limited to: poor bone quality, an increase in postoperative activities in the face of poorer balance, coordination and vision, which all contribute to falls and injuries. There is increasing interest in the complications of PSF and revision operation in the literature [1–9]. Moreover, it continues to be a devastating complication of total knee arthroplasty (TKA).

The treatment principles for periprosthetic fractures following TKA include maintaining alignment with rigid internal fixation, obtaining bone union and recovering sufficient painless range of motion of the knee through early exercise [3, 4]. The treatment options include conservative treatment, such as closed reduction and cast immobilization, and surgery, such as open reduction and internal fixation, intramedullary nailing, revision TKA using a longer stem, external fixation and arthrodesis with bone graft [3]. Unfortunately, stable fixation is difficult to achieve in many case because of the age and accompanying osteoporosis [3, 4]. Also, management of these fractures presents a significant challenge to most orthopedic surgeons because most do not have adequate clinical experience with this problem. There are plenty of reports available in the literature mentioning comparable treatment outcomes between the RISN and FLP for dealing with this complex problem after TKR including recent multiple systemic reviews and the latest meta-analysis by Mohit Bhandari et al. [23–27].

The purpose of this study is to analyze the clinical results and related complications of the femur plate system and the retrograde-inserted supracondylar nail in the treatment of PSF.

## Materials and methods

This study was approved by the hospital ethics committee at our institution, and all participants provided informed consent. We performed a retrospective study using collected data from arthroplasty databases. We had 42 cases of PSF proximal to posterior stabilized TKA between January 2005 and December 2009. Twenty-four cases of periprosthetic supracondylar femoral fractures were treated with the femur plating system. The femur plate system implants included the LISS DF (Synthes^®^, West Chester, PA, USA) in 17 cases and NCB^®^ plate (Zimmer^®^, Warsaw, IN, USA) in 7 cases. The other 18 cases of periprosthetic supracondylar femoral fractures were treated with the RISN (4CIS^®^, titanium supracondylar nail, Solco Biomedial, Seoul, Korea). The LP (locking plate) was used with the MIPPO technique with sliding of the plate proximally in a submuscular and extra-periosteal fashion and reduction preferably by indirect methods in almost all 18 cases. The RISN was inserted by using the previous skin incision and mini-arthrotomy from the open femoral box taking due precaution to avoid hyperextension deformity by avoiding the posterior starting entry point [24]. There were 40 female and 2 male patients. The mean age at the time of TKA was 67.2 years and at the fixation of fracture was 69.9 years. The TKA implants utilized included Scorpio^®^ (Stryker, Mahwah, NJ, USA) in 24 knees, Vanguard^®^ (Biomet, Warsaw, IN, USA) in 8 knees, Genesis II Oxinium^®^ (Smith and Nephew, Memphis, TN, USA) in six knees, Triathlon^®^ (Stryker, Mahwah, NJ, USA) in three knees, and Columbus^®^ (B. Braun Aesculap, Tuttingen, Germany) in one knee. The mean interval from TKA to the development of a fracture was 25.5 months (range 2 weeks–7 years). All patients had a history of trauma, and “slip/fall” was the most frequently observed mechanism of injury (two cases of traffic accidents, three cases of minor trauma, 37 cases of slip injuries).

We classified all fractures using the AO classification system, and this study cohort was divided into subgroups according to the AO classification (33-A1 subgroup, *n* = 17; 33-A2 subgroup, *n* = 8; 33-A3 subgroup, *n* = 17).

For cases without complications (such as cases without any delayed union/non-union/breakage of plates, broken screws, screw pull-out after the index operation, infection after placing the plate or nail, or any further surgical intervention in the form of I&D, falling after undergoing FP/RISN). We retrospectively compared the clinical results [age, time to injury, body mass index (BMI), bone mineral densitometry (BMD), operation time, knee society score (KSS), complication rate] between the FP and RISN group, and also compared the clinical results of each of the three subgroups to observe the differences in these subgroups. For cases with complications (cases without all of the above-mentioned technical as well as implant-related problems), the demographic, surgical, radiological and instrumental factors were surveyed to evaluate their effect on the complications following fracture fixation.

Categorical variables were analyzed using *χ*^2^ or Fisher’s exact test. Non-parametric variables were compared using the Mann-Whitney *U* test. Statistical significance was set at *p* < 0.05 and the confidence interval (CI) at 95 %. Statistical analysis was performed with SPSS version 18.0 for Windows (SPSS Inc., Chicago. Illinois).

## Results

A total of 42 patients were assessed at a mean 34.6-month follow-up (range from 17.2 to 76.7 months). All fractures were immobilized for 6.1 weeks on average in the FP group and 4.2 weeks in the RISN group (*p* = 0.129). This immobilization period was not determined by clinical symptoms or radiographs but primarily by the judgment of the surgeon who set the fracture union.

Clinical union was observed and full weight-bearing achieved at 44.4 weeks of the average mean total with the 49.8th postoperative week on average (13.3–191 week) in the FP group and the 38.3rd postoperative week (16–121.3 week) on average in the RISN group (*p* = 0.649, Table 1). Radiological union was also observed on the radiographs taken at the 6th postoperative month in all the uncomplicated (35 cases) cases of both groups (i.e., excluding the seven patients who had undergone complication and revision surgeries).

**Table 1:** Comparison of the clinical results between the FP and RISN group

	FP group (*n* = 24)	RISN group (*n* = 18)	*p* value
Operative time (min)	135.0 ± 31.9	125.0 ± 38.5	0.223
Immobilization period (weeks)	6.1 ± 4.1	4.2 ± 1.8	0.129
ROM (°)	95.8 ± 19.9	100.7 ± 18.4	0.573
ROM reduction (°)	11.1 ± 14.5	7.2 ± 14.7	0.364
Knee society score	77.2 ± 12.7	81.8 ± 8.7	0.379
KSS functional score	76.5 ± 14.5	80.6 ± 10.9	0.310
Time to union	49.8 ± 42.5	38.3 ± 25.5	0.649

**Fig. 1:**
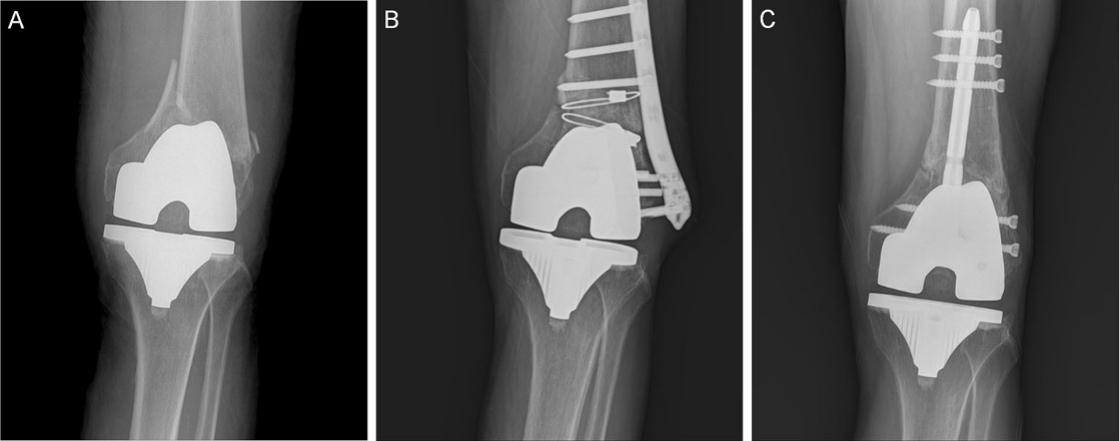
**a** Case 1. Preoperative X-rays of a periprosthetic fracture of the *left* knee in a 75-year-old man. **b** Radiograph of metal failure with the FP system taken 6 months later. **c** Follow-up radiograph taken 2 years later after fracture fixation with the RISN showing callus formation

**Fig. 2:**
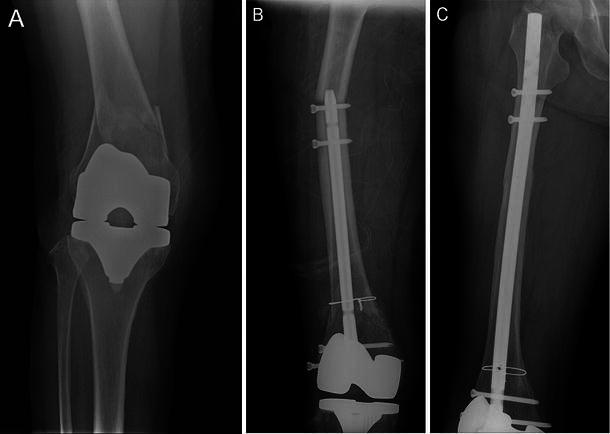
**a** Case 2. Preoperative X-rays of a periprosthetic fracture of the *right* knee in a 62-year-old woman. **b** A radiograph of refracture at the tip of the retrograde nail taken 3 months later after the RISN fixation. **c** Follow-up radiograph taken 2.5 years later after fracture fixation with a femoral intramedullary nail showing callus formation

At the latest follow-up of the uncomplicated cases, the mean range of motion (ROM) was 96.5° in the FP group (extension to flexion, 1.5° to 98°) and 105.7° in the RISN group (extension to flexion, 2° to 107.7°); the average ROM differences between before and after the fracture fixation were reduced by 11.1° in the FP group and 7.2° in the RISN group; the average knee score was 77.22 in the FP group and 81.81 in the RISN group; the average knee function score was 76.52 in the FP group and 80.63 in the RISN group, respectively. There were no significant differences between the two groups (*p* = 0.310, Table 1). Complications were seen in 29.2 % (7/24) of patients in the FP group, including five cases of nonunion with broken screws and two cases of nonunion with medial translation (mean 3.41 mm) due to screw pull out. These cases required additional surgical interventions including the Ilizarov method in one case and revision of plating in three, ultimately resulting in stiffness (mean ROM reduction −47.8°). In the RISN group, two cases of fracture at the level of the proximal nail tip and three cases of nonunion with varus deformity (mean 5.86°) (Tables 2, 3, 4) due to broken distal screws were seen (27.8 %, 5/18). Seven out of 42 patients underwent revision surgery at a mean interval of 46.4 weeks without any significant difference between the two groups (*p* = 0.512, Table 5). All seven cases with complications requiring additional revision procedures resulted in stiffness (mean ROM reduction −35°). The age at index TKA and fracture fixation was a significant risk factor for complications (odds ratio = 1.150, 95 % CI 1.01–1.30; odds ratio = 1.195, 95 % CI 1.02–1.39). No significant differences were found among the others factors (such as fracture type, instrument type, BMI, BMD, HTN, DM) between the two groups (Tables 6, 7); (Figs. 1, 2). None of the patients died during follow-up.

**Table 2:** Comparison of the clinical results between the FP and RISN group according to the AO classification (33-A1 subgroup)

	FP group (*n* = 10)	RISN group (*n* = 7)	*p* value
ROM (°)	93.0 ± 24.1	106.0 ± 14.2	0.143
Immobilization period (weeks)	5.6 ± 2.7	5.2 ± 2.4	0.404
Operation time (min)	129.0 ± 34.1	102.1 ± 11.5	0.043
Knee society score	83.2 ± 9.0	85.8 ± 2.2	0.735
KSS functional score	81.1 ± 10.8	75.0 ± 14.6	0.458

**Table 3:** Comparison of the clinical results between the FP and RISN group according to the AO classification (33-A2 subgroup)

	FP group (*n* = 4)	RISN group (*n* = 4)	*p* value
ROM (°)	96.7 ± 30.6	95.0 ± 17.3	0.857
Immobilization period (weeks)	8.5 ± 7.6	3.3 ± 2.1	0.229
Operation time (min)	112.5 ± 17.1	120.0 ± 14.7	0.486
Knee society score	69.5 ± 17.3	82.0 ± 8.5	0.248
KSS functional score	65.0 ± 23.8	87.5 ± 12.6	0.186

**Table 4:** Comparison of the clinical results between the FP and RISN group according to the AO classification (33-A3 subgroup)

	FP group (*n* = 10)	RISN group (*n* = 7)	*p* value
ROM (°)	98.0 ± 11.0	97.5 ± 10.6	0.829
Immobilization period (weeks)	5.2 ± 2.6	4.3 ± 1.9	0.587
Operation time (min)	147.0 ± 29.7	141.4 ± 51.3	0.807
Knee society score	74.9 ± 12.4	78.9 ± 11.2	0.617
KSS functional score	77.0 ± 11.8	80.7 ± 4.5	0.278

**Table 5:** Comparison of the failure results between the FP and RISN group

	FP group (four cases)	RISN group (three cases)	*p* value
Interval (second to final operation) (week)	39.6 ± 28.4	63.7 ± 61.6	0.512
Failure (case/total)	4/24	3/18	0.900

**Table 6:** Cross table of complication rate and fracture subgroup

Complication * AO classification, cross tabulation				
	AO classification			Total
	33-A1 subgroup	33-A2 subgroup	33-A3 subgroup	
Non-complication group				
Number	12	5	13	30
% of Total	28.6 %	11.9 %	31.0 %	71.4 %
Complication group				
Number	5	3	4	12
% of Total	11.9 %	7.1 %	9.5 %	28.6 %
Total				
Number	17	8	17	42
% of Total	40.5 %	19.0 %	40.5 %	100.0 %

This table showed no significant relationship between complication rate and fracture severity according to the AO classification (*p* = 0.767)

**Table 7:** Comparison of several risk factors between the complication and non-complication group

	Non-complication group (*n* = 30)	Complication group (*n* = 12)	*p* value
Immobilization period (weeks)	4.8 ± 2.4	7.0 ± 5.8	0.386
Age at the TKR	65.7 ± 6.5	71.0 ± 6.2	0.007
BMI	25.9 ± 3.8	26.8 ± 5.8	0.770
Time to injury from operation (months)	25.9 ± 26.4	24.6 ± 29.1	0.638
Age at fracture fixation	68.4 ± 6.6	74.5 ± 5.9	0.008
Operation time (min)	126.6 ± 33.1	141.8 ± 37.8	0.246
BMD	−2.7 ± 1.4	−2.5 ± 1.1	0.708

## Discussion

Fracture above the TKA is challenging to treat. PSF occurs more often in patients with compromised general health status and osteoporosis contributing to difficulties in obtaining solid fixation [5]. With the aging population, it is more likely to encounter patients who require TKA with known risk factors for PSF such as old age, poor bone stock, chronic use of corticosteroids, inflammatory arthropathy and stress risers—whether iatrogenic or due to local osteolysis, previous surgery, excessively stiff joints and various neurological conditions. Consequently, this is a heterogeneous group of patients, making the treatment results difficult to predict [6, 7].

Much work has been carried out on the management of femoral periprosthetic fractures, but less is known about their incidence, which ranges from 0.3 to 2.5 % after primary TKR and 1.6–38 % after revision procedures according to the reports [5, 6, 8]. The lower incidence of fracture after primary TKR compared with THR may explain the lack of a validated classification system to guide appropriate treatment [6]. The best operative technique remains somewhat controversial. Multiple factors must be considered before deciding on the treatment plan. These include the fracture pattern, degree of displacement and type of prosthesis used. The functional status of the prosthesis, including loosening, wear and instability, as well as the quality of the surrounding bone, must also be taken into account [9, 10]. The status of the patient, including medical comorbidities, is an additional important consideration [7, 10].

Several surgical options have evolved, including hybrid external fixation, intra-medullary nailing, conventional plates and locked plate fixation [7, 8, 11]. Less invasive stabilization systems offer advantages over conventional plates for the treatment of periprosthetic fractures associated with TKA [3, 12, 23]. These devices provide stable fixation in osteopenic bone, are adaptable to different types of fracture and prosthesis, and can be inserted by using a minimally invasive approach. These plates are particularly useful in the presence of an implant in the proximal femur as it allows uni-cortical screw fixation overlapping the distal part of the proximal implant, thus avoiding a stress riser between the two implants [3].

Retrograde intramedullary nailing was first introduced for the treatment of supracondylar fractures of the femur in 1991 and attempted in TKA patients in 1994 [13]. The technique is relatively simple to perform and enhances fracture healing by providing proper stability with minimal soft tissue stripping. However, its use is limited to the total knee prosthesis with an open-box design and when comminution of the distal femur is sufficiently minor to allow the stable insertion of at least two distal interlocking screws.

Several biomechanical studies have compared fixation techniques of supracondylar femur fractures proximal to the TKA [14–16]. Biomechanical comparison between the retrograde-inserted intramedullary nail and plate fixation in these fractures showed retrograde nails to be inferior in initial fixation to dynamic condylar screws and blade plates but superior to condylar buttress plates. The LISS showed greater elastic deformation but less permanent deformation than the other devices tested. Comparing the LISS and RISN biomechanically, the RISN may provide greater stability in patients with a posterior cruciate ligament-retaining femoral TKA component [14].

Historically, ORIF of these fractures with plates and screws has been plagued by significant rates of malunion and nonunion [7, 9, 10]. Zehntner and Ganz [17] successfully treated six patients with condylar buttress plates. Moran et al. [18] reported on 15 displaced fractures treated with ORIF; 10 of the 15 patients demonstrated good results. There were, however, two malunions and three nonunions. Retrograde intramedullary fixation and supracondylar nails have been proposed to improve the rate of union while decreasing soft-tissue trauma [9]. Mclaren et al. reported on seven cases treated with supracondylar nailing, with stable fixation achieved in all patients [19]. Jabczenski et al. [20] similarly treated four patients with an intramedullary reamed nail, and all four fractures healed without complications.

Despite the possible biomechanical superiority of the retrograde IMN for treatment of these injuries and good results being reported in small series in the literature, there are several ongoing concerns about using the retrograde intramedullary nail.

Some prosthetic designs prohibit the use of intramedullary fixation because of canal or notch mismatch with the nail diameter or the presence of a stemmed femoral component [10]. In dealing with a closed box, posteriorly stabilized knee or a notch vs. nail or canal diameter mismatch, a burr may be needed to enlarge the opening, thus introducing metal debris into the arthroplasty [5, 7].

Bezwada et al. [7] compared an open reduction group with a retrograde intramedullary nailing group. In their series, retrograde intramedullary nailing appeared to be the treatment of choice when feasible, and traditional ORIF may also yield satisfactory results in those designs that cannot accommodate retrograde intramedullary fixation.

Similarly, in our study, no significant differences were seen in the clinical results, time to clinical and radiological union, complication rate, or postoperative ROM between the FP and RISN group.

The reported complications after periprosthetic fracture fixation include nonunion, malunion, stiffness and infection [8]. Different complication rates have been reported. Herrera et al. [21] reported an overall nonunion rate of 9 %, failure of fixation in 4 %, infection rate of 3 % and revision surgery in 13 % with a different method of fixation. Studies using locking plate fixation reported a mean rate of nonunion of 5.3 % (1.8–14 %), of fixation failure of 3.5 % (0.9–12 %), of deep infection of 5.3 % (1.8–14 %) and of further procedures of 8.8 % (3.8–19 %) [21]. Large et al. [5] reported 17 % (5/29 pts) malunions with the LISS or condylar locking plate. Fulkerson et al. [22] reported 11.1 % (2/11 patients) nonunion, 5.5 % (1/11 patients) delayed union and 5.5 % (1/11 patients) infection.

In our study, the overall complication rate was 29.2 % (7/24) in the FP group and 27.8 % (5/18) in the RISN group, including nonunion with broken screws, screw pull-out, new fracture at the level of the proximal nail tip and stiffness. The complication rate in our study was higher than that in other studies. We think that this was due to inclusion of cases of relatively older patients and treatment by a number of surgeons with different levels of experience. The authors acknowledge the fact that this study represents a retrospective review of an uncommon fracture treated by a number of surgeons with different levels of experience in treating periprosthetic injuries, and this is a limitation of our study. Also, the risk factor analysis of complications was performed between the complication group versus non-complication group. More clarification would be provided by comparison in each instrumentation group, but we did not have a high enough number in each group. We will pursue our study to further clarify the risk factors for complications.

In our retrospective review, no significant differences were seen for several risk factors, including as past medical history (diabetes mellitus, hypertension), implant type for the TKA, bone mineral density, time to injury from the TKA operation, body mass index, implant type for fracture fixation and operation time, except for age, in the complicated c and non-complicated cases.

In their recent meta-analysis, Bhandari et al. concluded, “Locked plating and RIMN offer significant advantages over non-operative treatment and conventional (non-locked) plating techniques in the management of periprosthetic femur fractures above total knee arthroplasties. Locked plating demonstrated a trend towards increased nonunion rates when compared to RIMN. Malunion was significantly higher with RIMN compared to locked plating” [27]. Meek et al. [6] stated that female patients aged >70 should be warned of a significantly increased risk of periprosthetic fracture after hip or knee replacement. Risk of fracture is significantly high in patient >80 years [28].

## Conclusion

Although both groups showed significant clinical improvement following surgical treatment of PSF, the complication rates were significant. Fixation method and fracture type did not cause an increase in the complication rate, but there was a trend toward higher non-union rates with the FP method and higher refracture rates with the RISN method. In spite of similar clinical results between the two groups, each fixation method has its own limitations. While treating patients with several risk factors, especially old age, noting the fact that increasing age correlated with an increased complication rate, more careful attention should be paid to elderly patients in terms of both prevention and surgical care.
